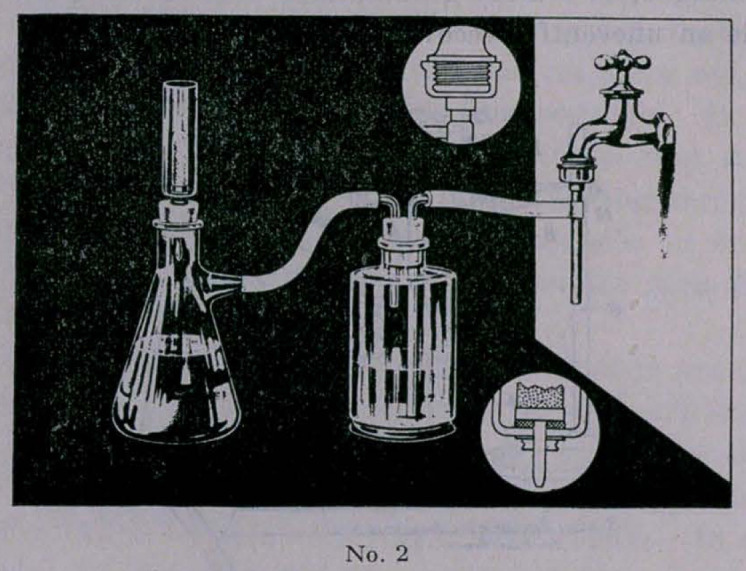# The Great Yellow Peril*Continued from September issue.

**Published:** 1916-10

**Authors:** Charles H. Duncan

**Affiliations:** New York City, N. Y.


### The Great Yellow Peril.*

BY CHARLES H. DUNCAN, M. D., NEW YORK CITY, N. Y.

The accompanying illustrations are of the Duncan Autotherapeutic Apparatus No. 1 and No. 2. No. 1 is designed for the field hospital, where there is no running water; the mixture of water and pus is poured in the cylinder No. 1, the atomizer bulb forces air on top of this, sending the fluid through filter No. 3 into the sterile retainer No. 4. The unmodified bacteria-free toxin complex is in the filtrate in No. 4 and ready for use.

Apparatus No. 2 is designed for the base hospitals. It is similar in construction to No. 1, with the exception that it has a syphon attached to water faucet, the water flowing through the syphon tends to create a suction on the underside to draw the soluble toxins through the Berkfeld filter, and the germ-free filtrate is found in the suction flask below.

Where respiratory infections are being treated, as in pneumonia, bronchitis, rhinitis, etc., gravity filtration is not sufficient, and we must resort to either the air pressure of No. 1 apparatus, or the suction of No. 2, to hurry the process of passing the mixture of water and exudate through the porous filter candle. In a military hospital but one filter and two dozen four-ounce bottles and corks is all that is required in addition to the other paraphernalia which the surgeon carries—for every ten beds. In reality, the only additional apparatus required to employ this method of treatment is a small gasoline stove and an agate pan 12 inches in diameter and 2 inches high for sterilization, a good hypodermic syringe, platinum needles, small bottle of iodine, toothpicks and cotton for the sterilization of the skin. Before being used, the porcelain part of the filter should be scrubbed lightly with a moderately soft brush, preferably, under running water; then all parts of the filter should be boiled for a half hour. It is needless possibly to add that the syringe and needles should be sterilized after each treatment. This is fast becoming the method of wound treatment in all parts of the world, and it is only a question of time when it will be universally employed by all physicians.

*Continued from September issue.

The following cases show conclusively that a diagnosis is often unnecessary, as far as a cure is concerned:

CASE 1.—Patient female, age 36, accidentally ran a hat pin deep into her wrist. Four days afterward when she applied for treatment her temperature was 103° F. The lymphatics were streaked up past the elbow. The patient was in a nervous state, and complained of severe pain in the forearm. The arm was opened under anesthesia at seat of puncture, but no pus was found. Within twenty-four hours a thin serous exudate appeared on the gauze, which proved to be streptococcus. No bandage was placed on the wound, but the patient was instructed to lick it whenever there was irritation in it. Within twenty-four hours the discharge ceased and all symptoms subsided. The patient was then discharged, as healthy granulations were covering the wound. She made an uneventful recovery.

No. 1

CASE 2.—Patient, boy 16 years old, fell off a wall, cutting a gash through the scalp two inches long. The hair was clipped with household scissors, and the flaps loosely draw together with a domestic needle and thread. Nothing was sterilized, not even the operator's hands, unsterile gauze dressing was placed over the wound; this was removed in twelve hours and the stained part was cut with scissors and placed in a bottle with two ounces of tap water. This was thoroughly shaken and the decanted fluid was given the patient to drink. This was done twice daily for three days, and then once daily for two or three days afterwards. The wound apparently healed by first intention.

CASE 3.—Dr. H. T. B. while fishing had a hook imbedded in the hand by another fisherman. The hook was cut out with an unsterile fish knife. He sucked the wound conscientiously for two or three days when there was irritation in the parts. The wound apparently healed by first intention.

CASE 4.—Patient female, age 26, large furuncle, staphylococcus pyogenes aurius. Twenty minims of the filtrate injected. Healed in twenty-four hours.

CASE 5.—J. R. Mansfield, M. D., of Philadelphia, reports the following cases treated autotherapeutically: "While treating a patient suffering with mastoiditis in St. Luke's Hospital autotherapeutically, I was invited by the attending physician and pathologist to step in a private room and look at a patient suffering with a stubborn infection of the scalp. A month previous he was thrown from an automobile. His scalp was torn and pushed

No. 2

back over a large area in the parietal region. In spite of all that apparently could be done the infection persistently grew worse. The patient was very weak and the blood count showed that unless something was done quickly the case would terminate fatally. The physicians were much concerned and asked my advice. I took a medicine dropper from my case and drew into it about ten drops of pus. This was placed in four ounces of water; of this the patient was given a teaspoonful every hour for eight doses, beginning at 12 m. At 10 p. m. that day the interne failed to discover any more pus. The next morning when the pathologist saw the case said, 'I would not have believed it had I not seen it with my own eyes. I was afraid yesterday you would poison the patient.' The mastoiditis case previously referred to was cured quickly by means of autotherapy and operation aborted."

Case 6.—Patient male, age 45 years, had a severe infection of the middle finger on the left hand. He was treated by two eminent surgeons of New York City, with the result that after three weeks' treatment they informed him his finger would have to be operated again and possibly amputated. He was referred to the writer by a veterinary who is a most enthusiastic autotherapeutist from the marked successes he is having with this method of therapy in the treatment of animals, and by a patient whose brother had been given up by the attending surgeon to die of septic poisoning, but who was cured quickly by autotherapy. Patient was received at 10 a. m.. At 2 p. m. a hypodermic injection of the unmodified toxin complex was given. At 6 p. m. the pain stopped and the patient slept better that night than he had in three weeks. In twenty-four hours the pus had disappeared; in three days the patient had so far recovered that he was discharged in the custody of his wife, who is a trained nurse. Two months later this patient placed a series of reprints of my former articles dealing with the technic of autotherapy in its application to bronchial conditions and pus infections in the hands of the surgeon general of the French army. He showed him his finger. He told him of the almost miraculous cure that was made by means of autotherapy. He was the object of much interest, as he was the first patient treated by Dr. Duncan himself the French surgeon general had ever seen, although he was familiar with autotherapy from an article that had appeared in the *Paris Medical* just previous to the outbreak of the war. He stated "autotherapy is being used successfully in many hospitals in France. So successfully, in fact, that accounts of the quick cures made by autotherapy were published in a number of the French daily papers."

BIBLIOGRAPHY.

1. "A New Method of Vaccine Treatment and Prevention of Sepsis" (*Medical Record*, September 16, 1911), Chas. H. Duncan.
2. "Autotherapy" (*New York Medical Journal*, December 14 and 21, 1912), Chas. H. Duncan.
3. "Autotherapy" (*Veterinary Journal*, London, October, 1912), D. J. Mangen.
4. "Autotherapy in the Prevention and Cure of Purulent Infections" (London, *Practitioner*, April, 1914).
5. "Prevention and Treatment of Septic Wounds in Warfare" (*Indian Medical Gazette*, November, 1914), F. W. Sumner.
6. "A Positive Method of Curing Purulent Infection"; An Appeal to the Army Surgeon (*Interstate Medical Journal*, October, 1915), Chas. H. Duncan.
7. "A Positive Method of Preventing and Curing Purulent Infection"; An Appeal to the Army Surgeon (*American Medicine*, October, 1915).
8. "Autotherapy Versus Operations" (*The American Practitioner*, July, 1913), Chas. H. Duncan.

9. "Autotherapy in Surgery" (*American Journal of Surgery,* October, 1913), Chas. H. Duncan.
10. "Autotherapy" (*Practical Medicine,* Delhi, India, July, 1914), Chas. H. Duncan.

## Figures and Tables

**No. 1. f1:**
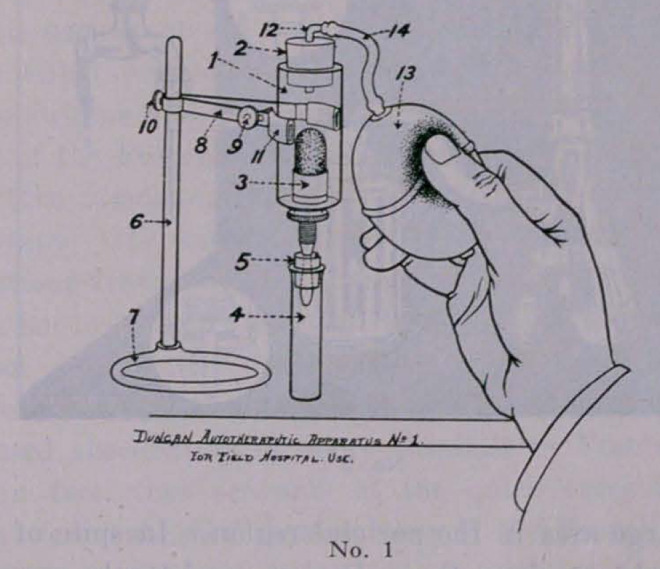


**No. 2. f2:**